# Benefits and Risks of Sun Exposure to Maintain Adequate Vitamin D Levels

**DOI:** 10.7759/cureus.38578

**Published:** 2023-05-05

**Authors:** Jonathan R Raymond-Lezman, Suzanne I Riskin

**Affiliations:** 1 Foundational Sciences, Nova Southeastern University Dr. Kiran C. Patel College of Osteopathic Medicine, Clearwater, USA; 2 Department of Internal Medicine, Foundational Sciences, Nova Southeastern University Dr. Kiran C. Patel College of Osteopathic Medicine, Clearwater, USA

**Keywords:** uv exposure, sunscreen, spf, sun protection factor, melanoma, cancer, hypovitaminosis d, deficiency, vitamin d

## Abstract

Vitamin D is a hormone that can be generated in the skin upon ultraviolet light exposure or ingested through supplementation. Vitamin D deficiency may have numerous deleterious effects on health. Sun avoidance strategies should be avoided due to the unwanted health risks associated with hypovitaminosis D. We present an objective investigation of the benefits and risks of using sun exposure to increase vitamin D levels and how it impacts human health. A review of the literature was conducted using Embase and PubMed to examine the relationship between UV exposure, vitamin D levels, health benefits, and risks.

UV exposure is the primary method of boosting serum vitamin D levels, which accounts for numerous health benefits. Higher levels of vitamin D are associated with protection against cancer development, including melanoma. Latitude, season, skin color, and sun protection determine UV absorption and vitamin D production. Public health sun protection guidelines decrease the incidence of skin cancer, but sun avoidance can cause hypovitaminosis D. Serum vitamin D levels less than 16 nmol/L increase morbidity through increased non-cutaneous disease. Sun protection strategies should still be implemented to reduce skin cancer, and sunscreen only minimally lowers vitamin D production. Vitamin D deficiency can increase chronic diseases and cancer, while adequate vitamin D levels can help prevent them. UV exposure and vitamin D production are dependent on many factors. Increasing UV exposure without causing sunburn maximizes vitamin D production.

## Introduction and background

It is well known that “getting some sun” increases vitamin D production [1,2]. Less is known about quantifying accurate amounts of ultraviolet radiation exposure that achieve the positive benefits of increased serum vitamin D while minimizing skin cancer risk. Vitamin D exerts its effects through multiple mechanisms that aid in tissue repair, downregulate harmful inflammatory mediators, and prevention of various cancers and their proliferation [3-5].

Endogenous vitamin D can be easily produced through ultraviolet light exposure. Vitamin D supplements also safely increase serum vitamin D levels, but not as effectively or rapidly as using sunlight. Only a small amount of exposed skin is needed to produce vitamin D. Maintaining higher serum vitamin D levels improves the prognosis of multiple systemic diseases, but it is difficult to determine how much is needed due to factors influencing absorption [1-5].

## Review

The goals of this review were to summarize relevant published research regarding vitamin D, its benefits and adverse effects, the use of ultraviolet light to synthesize vitamin D, supplementation when endogenous vitamin D is not possible, and their relation to one another. This review mainly focuses on vitamin D deficiency along with vitamin D’s role in morbidity and mortality. This review also highlights areas that need further research.

Search strategy

**Figure 1:**
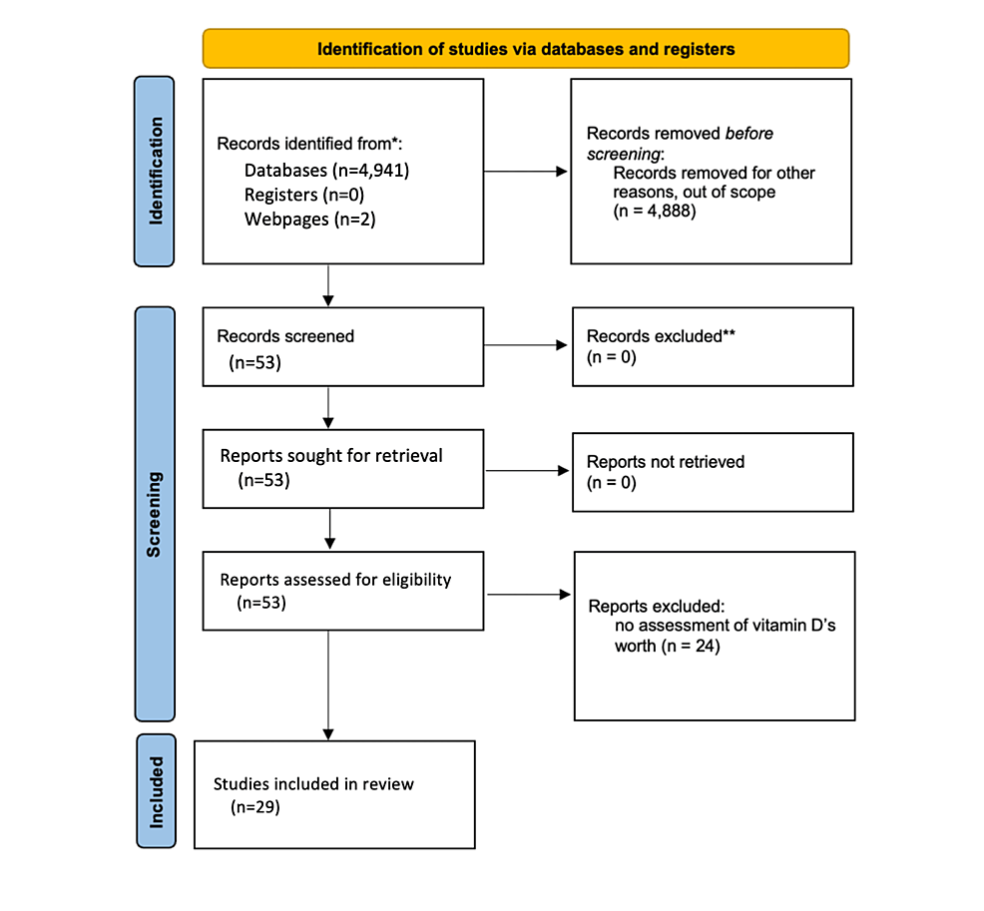
PRISMA diagram to determine studies used in benefits and risks of sun exposure to maintain adequate vitamin D levels review. PRISMA: Preferred Reporting Items for Systematic Reviews and Meta-Analyses.

PubMed and Embase were used as databases for peer-reviewed articles. Within Embase, the terms “vitamin D,” “melanoma AND vitamin D,” “UV AND vitamin D,” and “sunlight AND vitamin D” were used. The use of Boolean terms was to narrow searches to maintain articles within the scope of this review. In PubMed, the terms “vitamin D” and “vitamin D deficiency” were used. The Food and Drug Administration’s (FDA) webpage was utilized to include dietary recommendations for vitamin D. The Center for Disease Control’s webpage was utilized to indicate sun-protective recommendations. Inclusion criteria were studies conducted that included ultraviolet light's influence on vitamin D, the role of vitamin D in the pathogenesis of cutaneous diseases and those of other organ systems, and outcomes pertaining to hypervitaminosis D and hypovitaminosis D. Furthermore, current guidelines for serum vitamin D levels and the outcomes vitamin D may have had on patients with COVID-19 infection were also used. Exclusion criteria were for articles that did not contain pathologic outcomes or hypotheses due to serum vitamin D levels. These searches generated 4,936 articles. After inclusion and exclusion criteria were implemented, a total of 29 articles were used in this review. Figure 1 demonstrates the selection of articles used in this review based on the inclusion and exclusion criteria.

Vitamin D synthesis and mechanisms of action

Endogenous vitamin D from UV exposure accounts for over 80% of the total vitamin D produced [1,2]. Epidermal keratinocytes contain the vitamin D precursor 7-DHC. Ultraviolet-B (UVB) radiation, a shorter wavelength, opens the B ring of the steroid and forms previtamin D3. After thermal isomerization to form cholecalciferol, the molecule is then called vitamin D3. Two separate hydroxylation reactions occur to create the active form of vitamin D, 1,25-dihydroxyvitamin D (1,25(OH)D). The first hydroxylation reaction occurs in the liver and produces 25-hydroxyvitamin D (25(OH)D), which can be measured in the serum to determine vitamin D status. The second hydroxylation reaction primarily occurs in the kidneys, but many other cell types, such as skin cells, use the same enzymes to produce 1,25(OH)D [3]. The kidneys facilitate the uptake of 25(OH)D3 using the vitamin D-binding protein. As the glomerular filtration rate decreases due to chronic kidney disease, serum vitamin D levels also decrease [4].

After sunlight exposure and previtamin D3 are produced, additional UVB radiation is isomerized into two other products: lumisterol3 and tachysterol3. These two products have no impact on calcium homeostasis. Upon exposure to UV radiation, the skin can convert around 15% of 7-dehydrocholesterol into previtamin D3. Any other radiation absorbed is converted into lumisterol 3 or tachysterol 3. While these products are being produced, if more UV exposure occurs then suprasterols and toxisterols are produced. Since none of these products have calcium-modulating activity, it validates safe vitamin D production through UV exposure. Any additional previtamin D3 produced is simply degraded. These degraded photoproducts can be beneficial for tumor agenesis and prevention. Lumisterol 3 is converted to 1,25-dihydroxylumisterol 3, which may have anti-tumor effects on the skin. Some suprasterols exhibit antiproliferative effects on keratinocytes. Therefore, cutaneous production of vitamin D3 may have many more benefits from its isomerized degradation products than ingestion of vitamin D3 as a dietary supplement [5].

Endogenously produced 1,25(OH)D maintains calcium homeostasis [3,6]. Osteoblasts require ions to synthesize collagen matrix, and vitamin D maintains the calcium-phosphate balance to achieve this. Deficiencies in vitamin D are noted with high parathyroid hormone levels, which function to maintain serum and bone calcium balance. Parathyroid hormone pulls calcium off bones to release into the blood and dumps phosphorus into the urine. On the other hand, hypervitaminosis D can lead to hypercalciuria and hypercalcemia, with weakness, headaches, confusion, and polyuria manifesting as some common symptoms. Such high amounts of vitamin D ingestion are needed to achieve hypervitaminosis D that toxicity is rare. This is due to the final regulatory mechanism the kidneys perform in hydroxylation [6].

Vitamin D asserts its effects by binding to the nuclear vitamin D receptor (VDR). These are distributed in most human tissues. In vitro within the epidermis, 1,25(OH)D acting through the VDR reduces the proliferation of keratinocytes and melanocytes and promotes differentiation and migration into the epidermis [3]. VDR functions as a tumor suppressor, and a decrease in its expression is associated with an increase in malignant melanoma progression. CYP11A1 derivatives regulate skin functions through retinoic-acid orphan receptors (ROR) alpha and gamma expressed within the skin. Nonclassical vitamin D3 hydroxyderivatives, 20(OH)D3 and 20,23(OH)2D3 can act as weak RORa and RORy inverse agonists. These enable tumor suppression and anti-inflammatory effects. The classical 1,25(OH)2D3 and CYP11A1 D3 derivatives act as aryl hydrocarbon receptor (AhR) and liver X receptor agonists to regulate cellular proliferation, inflammation, and melanogenesis. Furthermore, the AhR can downregulate proinflammatory responses, antioxidation, and detoxification. 1,25(OH)2D3 downregulates many pro-inflammatory cytokines such as interferon-gamma, TNF-α, Th1, Th2, and Th17 while increasing anti-inflammatory cytokines such as IL-10 and Tregs. Higher levels of IL-10 reduce mast cell-mediated allergic responses and IgE conversion [7]. By increasing interleukin 10 (IL-10), the Th1 and Th2 responses also increase, dendritic cell maturation is inhibited, and CD4^+^/CD25^+^/Foxp3 cytotoxic T-lymphocytes are induced [8].

Finally, vitamin D has many properties that allow it to increase differentiation, maturation, and senescence in normal and malignant cells. It can inhibit the G0/G1 and G2/M phases, downregulate Bcl-2 and Bcl-XL anti-apoptotic proteins to induce cell death and increase pro-apoptotic proteins such as Bcl-2-associated X protein (BAX), Fas-associated death domain (FADD), and caspases. Vitamin D also reduces vascular endothelial growth factor (VEGF) and inhibits angiogenesis [9].

There are multiple factors that can reduce vitamin D production. Skin color, qualitatively measured using the Fitzpatrick scale, may have detrimental effects of maintaining vitamin D production. As the Fitzpatrick type increases on the scale from I to VI, going from lighter skin to darker skin, vitamin D production decreases. This suggests that darker skin requires larger doses of UV radiation to generate the same amount of vitamin D that lighter skin generates. Higher melanin level, expressed in darker skin as compared to lighter skin, provides protection from UV radiation [10]. Fitzpatrick scale types I and II have lower minimal erythema doses, the amount of UV radiation needed to induce sunburn, compared to types V and VI. This allows higher amounts of UV penetration and higher amounts of vitamin D production for types I and II skin. However, it also increases the risk of developing skin cancer and other UV-induced dermatoses [2]. Darkly pigmented groups of individuals may suffer from vitamin D deficiency due to melanin competing with 7-DHC for UV absorption [6]. Individuals with lighter skin can generate >50 nmol/L of 25(OH)D from 30 minutes of sun exposure daily, but darker skin requires upwards of two hours of exposure to reach the same amount produced [2].

Wacker and Holick conducted a study that compared Fitzpatrick skin types in relation to vitamin D production using UV tanning beds. They found that when exposed to UV light from a tanning bed, those with Fitzpatrick type II skin increased their rate of vitamin D production by 30 times the rate of those with Fitzpatrick type V skin [5]. The type II skin demonstrated no significant increase in vitamin D production unless it was exposed to five times the amount of radiation as before the UV bed was introduced. Upon secondary exposure, the type II skin generated 15 times the amount of vitamin D it previously did. Another study surgically removed dark and light skin and placed the samples underneath sunlight in Boston, Massachusetts. The study demonstrated that after 30 minutes of exposure, the lighter skin converted 3% of cutaneous 7-dehydrocholesterol into previtamin D3, whereas the darker skin only converted 0.3% [5].

Clothing and UV avoidance also decrease vitamin D production. A study conducted by Alagöl et al. involved Turkish women who covered most of their bodies in clothing due to cultural tradition. The results showed a severe deficiency in vitamin D compared to women who were exposed to more of their skin [11]. 

Production of vitamin D3 is dependent on UVB penetrance and absorption [3]. Geographic location is a considerable factor when determining the need for increased vitamin D in at-risk populations. The solar zenith angle (SZA) is the angle the sun in the sky makes compared to a latitude-specific vertical angle. A small SZA around noon in the summer has the highest atmospheric UV penetrance [2,3]. However, at latitudes greater than 50 degrees, virtually no vitamin D is produced during the winter and spring regardless of skin type. Cloud cover also diminishes UV absorption resulting in hypovitaminosis D. During the industrial revolution, rickets incidence increased as pollution increased. A study in Delhi also demonstrated lower levels of 25(OH)D in children as pollution increased. In the United Kingdom, vitamin D deficiency is highest among adolescent females, potentially due to less time spent outdoors [12]. Natural light coming through windows did not demonstrate the ability to jumpstart vitamin D synthesis. Moreover, indoor UV tanning beds did increase vitamin D production but are not recommended due to the higher risk of skin cancer and dermatoses [2].

To better determine if seasonality and latitude can impact serum vitamin D levels, Godar et al. conducted a study that followed 2,000 children in the U.S. who lived in different geographic areas and exhibited various Fitzpatrick skin types. Their results indicated that only children at southern latitudes sufficiently produced vitamin D if they were Fitzpatrick type II. Children in northern latitudes failed to produce the same amount of vitamin D during the summer compared to children in southern latitudes. However, southern latitude children only produced an additional 10-20% of vitamin D than their northern counterparts, so summer UV exposure is still effective at generating vitamin D in the north. Neither group produced adequate vitamin D in the winter [10]. Vitamin D production is at its maximum at one-third of the minimal erythema dose. Once UV exposure increases to one minimal erythema dose, vitamin D synthesis halts [13].

Altitude impacts vitamin D synthesis dramatically. A study that compared vitamin D3 synthesis in vitro was conducted at various locations with various altitudes above sea level. Agra (169 m), Kathmandu (1400 m), and Mount Everest base camp (5,300 m) are in India at 27 degrees north latitude. It was observed that as altitude increased, vitamin D production also increased. The vitamin D produced at the Mount Everest base camp was five times greater than that produced at Agra. This phenomenon is due to the relative thickness of the troposphere at various altitudes. Higher altitudes have less atmosphere to absorb UV radiation, thus allowing higher amounts of UV radiation to penetrate the skin [5].

Studies have shown the efficacy of sunscreen in the production of erythema, UV-induced immunosuppression, and DNA-pyrimidine dimers [14,15]. Sunscreen also protects against squamous cell carcinoma, melanoma, and photoaging. It does not protect against basal cell carcinoma. The sun protection factor (SPF) is a value set that limits UVB exposure and erythema but does not correlate with ultraviolet-A (UVA) prevention. Specific broad-spectrum sunscreen is needed to ensure protection against UVA radiation, which is more abundant than UVB and causes photoaging and immunosuppression [15].

Since solar UVB exposure increases vitamin D production, it may be thought that the use of sunscreen greatly inhibits vitamin D status. Several reviews of sunscreen use and vitamin D synthesis have shown that this is not the case [15]. Sunscreen decreases UV exposure and decreases vitamin D production. However, even with the sun protection factor, 15.6% of the UV radiation still penetrates the skin and is absorbed. In a study comparing individuals who utilized sunscreen regularly and those who did not, sunscreen users were not vitamin D deficient as compared to the control group. Using sunscreen daily reduces the risk of melanoma and non-melanoma skin cancer, aging, and immunosuppression while maintaining vitamin D levels [6]. However, less is known about application strategy. The recommended amount of sunscreen applied is 2 g/cm^2^, but a study performed in Egypt that followed vacationers determined their average application was only 0.79 g/cm^2^. This leaves questions regarding sunscreen’s impact on vitamin D status, as application density has not always been known in previous studies [15].

A study by Young et al. was conducted to determine if sunscreen negatively impacts 25(OH)D production. Participants vacationing in Tenerife, which has a similar latitude to Florida in the US, were placed into one of four categories. The first group, a control, stayed at home in Poland and did not go on the trip. A second group was instructed to use sunscreen at their own discretion. A third group was given SPF 15 with low UVA protection. Finally, a fourth group was given SPF 15 with high UVA protection. These last two groups were instructed to reapply frequently. After body surface exposure was normalized for all groups, the two groups given SPF 15 and frequent reapplication did not show any erythema. The discretionary group did show erythema [16].

When 25(OH)D serum levels were measured, the control group in Poland declined by 2.5 ± 5.6 nmol L^−1^. This drop in 25(OH)D indicates that food or exogenous vitamin D does not contribute to serum vitamin D levels or that the food consumed was not vitamin D fortified. Overall, the lack of UV exposure in the control group demonstrated a positive correlation between the levels of vitamin D compared throughout the experiment [16].

The three sunscreen groups showed improved 25(OH)D levels post-vacation. The discretionary group increased by 28.0 ± 16.5 nmol L^−1^, but with sunburn, they increased their risk of developing melanoma. The high UVA protection group increased by 25(OH)D 19.0 ± 14.2 as compared to the low UVA protection group at 13.0 ± 11.4 nmol L^−1^. By increasing UVA protection, UVB radiation can penetrate the skin deeper and induce higher endogenous vitamin D production. This study quantifies the benefits of UV exposure while using sunscreen. By utilizing SPF when encountering high amounts of UV exposure, erythema and dermatoses are protected against, but endogenous production of vitamin D is unaffected [16].

To quantify vitamin D production with the use of sunscreen, Marks et al. performed a study in Australia at 37 degrees of latitude, similar to Florida, to determine if sunscreen inhibited vitamin D production. It was demonstrated that by using SPF 17, participants were able to increase >7 nmol/L of vitamin D levels while preventing the development of actinic keratoses. Other studies have also confirmed that sunscreen alone does not cause vitamin D deficiency. Overall, while endogenous vitamin D from UV exposure is the most advantageous method of producing vitamin D, the increased risk of skin cancer and photoaging prompt regular sunscreen use and skin wellness visits [6,17].

It has been suggested by Passeron that healthy individuals should utilize sunscreen broad-spectrum high UVA protection to attenuate UVB radiation optimally. For individuals with photodermatoses, physical barriers and sunscreen should be used, and further vitamin D supplementation may be needed. Little is also known about the correct minimal UVB exposure based on latitude, time of day, body surface area, and Fitzpatrick skin type to effectively recommend exposure limits [15].

Ultraviolet light exposure, skin, and vitamin D

In the 1920s, Coco Chanel created a new “tanned is beautiful” campaign that promoted the use of tanning oils during UV exposure. It later caught on that using oils in the sun would “promote health” and vitamin D production. Furthermore, it was thought that if people consumed vitamin D from cod liver oil to prevent rickets, then more vitamin D from sun exposure would be increasingly beneficial. Exaggerated health claims surrounded these products, but the claims were ill-advised and potentially drawn from financial benefit. Since the 1890s, medical literature cited UV exposure as a carcinogen. By the 1960s, UV-induced DNA mutations were characterized. In the 1990s, signaling pathways and genes were identified that targeted mutation. Despite one in 70 people in the United States developing malignant melanoma within their lifetime, with 8,000 deaths annually, the trend is that being tan is considered a healthier appearance and is still sought after [6].

In 1941, Frank Apperly, a pathologist, published a study indicating an inverse relationship between levels of UV radiation exposure and cancer mortality in the United States. He stated, “the presence of skin cancer is really only an occasional accompaniment of a relative cancer immunity in some way related to exposure to ultraviolet radiation.” Since his study, additional studies confirm that living closer to the equator lowers the risk of internal organ cancer death. In 2005, Berwick et al. concluded that the presence of solar elastoses, high amounts of sun exposure, and sunburns were inversely proportional to death from melanoma [1,18].

UV exposure is implicated in many skin conditions, but the only benefit indicated is the production of vitamin D. The Center for Disease Control encourages minimal time spent in the sun while wearing maximum protective clothing and sunscreen [19]. This effectively reduces the incidence of skin cancer when adhered to properly, but fails to address the array of health problems that a lack of vitamin D can cause [20]. UV exposure causes basal cell carcinoma, squamous cell carcinoma, malignant melanoma, and among others. Short, intense periods of UV exposure causing sunburn, especially during childhood, greatly increases the risk of developing melanoma, while chronic UV exposure is linked to squamous cell carcinoma and actinic keratosis. UVA radiation travels deep into the dermis and causes skin aging, whereas UVB radiation stays within the epidermis and causes burns [1].

Spath et al. conducted a study comparing in vitro measurements of 1α-OH-D3 along with measurements of malignant melanoma metastasis. The study stated that higher levels of 1α-OH-D3 impaired malignant melanoma cell proliferation-inducing cell cycle arrest. This was correlated with an increase in cyclin-dependent kinase inhibitors, p21 and p27, which downregulated cyclin-D1. Apoptosis was not observed in the study; however, the authors noted that daily administration of 1α-OH-D3 inhibited malignant melanoma growth in vivo [9].

The relationship between vitamin D and melanoma is complex and not fully understood. In non-UV-exposed regions of the body, melanoma still develops. This could be from systemic immunosuppression or low serum vitamin D resulting in unopposed carcinogenesis. Melanocytes have reduced capacity for regeneration, proliferation, and DNA repair as compared to keratinocytes. These differences help differentiate the behavior between malignant melanoma and squamous cell carcinoma. It is important to evaluate the role of vitamin D in non-sun-exposed areas to better determine the relationship vitamin D’s protective benefits offer against melanoma [9].

To determine the influence vitamin D may have on the prognosis of patients with melanoma, out of 87 patients with malignant melanoma who were observed in a study, only 11 patients (12.7%) demonstrated normal serum vitamin D levels regardless of the anatomical location of the melanoma. Thus, it is hypothesized that vitamin D levels protect against melanoma. There have been many observational studies performed that compare vitamin D levels to malignant melanoma outcomes. It has been proposed by Slominski et al. to increase vitamin D intake by 10,000 IU daily or 50,000 IU weekly for patients with stage III or IV melanoma. Further studies are needed to evaluate the mechanisms behind vitamin D and malignant melanoma. More data would indicate if melanoma patients should be advised to increase their vitamin D levels by ingesting vitamin D supplements or to simply increase their UV exposure [9].

Alpha-Tocopherol, Beta-Carotene Cancer Prevention Study performed a case-control study that indicated there was no association between 25(OH)D levels and the development of cutaneous malignant melanoma (CMM). However, two subsequent studies showed that high levels of 25(OH)D at baseline were associated with a higher incidence of CMM and basal cell carcinoma (BCC). This indicates that 25(OH)D levels may be a surrogate for sun exposure. By increasing sun exposure, the risk of developing CMM and BCC also increases. Two meta-analyses recently published also supported these findings. Few studies have been conducted relating the exogenous intake of vitamin D supplements with the development of CMM. A recent meta-analysis indicates there is no association between the risk of CMM and vitamin D intake from supplements [3].

Although higher levels of 25(OH)D are correlated with the increased incidence of CMM and other keratinocyte cancers, the prognosis of malignant melanoma is better when 25(OH)D levels are higher. Serum 25(OH)D in patients with CMM is taken a few months after the diagnosis. Studies comparing 25(OH)D levels and CMM prognosis have shown that lower 25(OH)D is associated with thicker melanoma lesions, later staging, and a worse prognosis. More studies gathering serum 25(OH)D at the time of diagnosis are needed to further validate these findings. There is no evidence that vitamin D supplementation post-diagnosis aids in the prognosis of CMM. A few observational studies indicated that self-reported sun exposure reduced CMM mortality. Serum 25(OH)D levels were not obtained, which leaves these studies up to question. However, they further the need for additional studies to be conducted that measure 25(OH)D, compare sun exposure and identify the risk of CMM and keratinocyte cancer [3].

Hutchinson et al. found in a prospective study measuring serum 25(OH)D levels for patients newly diagnosed with malignant melanoma protected Breslow thickness and independent protection against relapse and survival. Since serum 25(OH)D was obtained shortly after diagnosis, it can be inferred that the patients already had higher levels of vitamin D when malignant melanoma developed. This indicates a protective effect against the early stages of melanoma. More research is needed to determine the protective benefits of vitamin D before, during, and after melanoma diagnosis and resection to determine this study’s validity [21,22].

There is research indicating that ultraviolet radiation (UVR) can be used to treat various pathogens such as Plasmodium spp., *Leishmania donovani infantum*, *Staphylococcus aureus*, *Mycobacterium leprae*, and *Malassezia furfur*. The effects of UVR on bacteria include directly altering replication, indirectly affecting defense mechanisms, or generating a more robust immune response. For viruses, systemic viruses like influenza and human immunodeficiency virus seem to react well to UV radiation, but cutaneous viruses like herpes simplex virus and human papillomavirus react poorly. The mechanisms of UVR on viruses are poorly understood, and it may just increase the immune system’s response. A study conducted between 2010 and 2018 regarding the influenza virus outbreak in Northern Europe concluded that incidence was higher when serum vitamin D levels were lower. This may indicate that UVR and vitamin D levels have heterogeneous effects on viral infection. The importance increases when characterizing treatment plans for chronic viral infections. UV radiation induces local immunosuppression by inducing cyclobutane pyrimidine dimers and 6,4-pyrimidine-pyrimidone dimers, keratinocyte lipid oxidation, and cytokine induction [23].

Skin aging occurs over time and is due to genetic, extrinsic, intrinsic, and environmental factors. Internally, hormones decline as humans age, and gene expression also changes. Skin aging is associated with immunologic dysregulation with imbalances between anti-inflammatory and pro-inflammatory responses. This leads to a chronic low-inflammation state, which results in impaired skin remodeling. Keratinocytes, melanocytes, and fibroblasts decrease regeneration due to senescence. The highest risk factors for premature aging due to external stressors include ultraviolet radiation exposure, smoking, and ambient pollutants. Continuous exposure to these substances increases radical oxygen species (ROS) and damages cellular DNA. This leads to impaired epidermal barrier function, altered skin microflora, and increased morbidity [24].

Observational studies have linked vitamin D and eczema. As latitude increases, vitamin D levels decrease, and eczema incidence increases. Symptom severity has also been observed to inversely correlate with vitamin D levels indicating a multifactorial response vitamin D has on eczema. Furthermore, new phenotypic studies show various vitamin D pathways are associated with eczema. Vitamin D may have the ability to regulate the genetic factors that predispose individuals to eczema, such as dysregulated immune system status and defective skin barriers like cathelicidins. In vitro studies have demonstrated suppression of *Staphylococcus aureus *through vitamin D metabolites, increasing keratinocyte production of cathelicidins. A few studies have been conducted measuring fetal vitamin D and comparing atopic dermatitis incidence within the first few years of life. Goldring et al. and Miyake et al. both concluded that there were no significant changes in eczema incidence when comparing various maternal vitamin D supplementations. These two studies should be read with caution as they failed to systematically measure maternal, fetal, umbilical, and amniotic sampling of vitamin D levels. They did find that latitude was a determinant in vitamin D status. They discovered that the higher the latitude, the lower the vitamin D levels within the umbilical cord [8].

The role of genotype in eczema and vitamin D metabolism further advances our understanding of eczema and vitamin D’s relationship. Liu et al. identified the L4, MS4A2, FCER1G, and CYP24A1 genotypes involved in IgE production, vitamin D metabolism, and food sensitization. An additional study showed low vitamin D levels at birth combined with a mutation in rs2243250 carrying a C allele was associated with three times increase in food sensitivity [8].

More studies are needed to correctly identify genes, haplotypes, predispositions, and potential remedies for atopic dermatitis using vitamin D. Many studies are observational and small leading to potential errors in significance. While there have been studies indicating UV exposure may increase atopic dermatitis in sun-exposed areas, the effects of nitric oxide (NO) and vasoactive substances on the skin make photodermatoses difficult to evaluate using ultraviolet radiation [8].

UV light and non-cutaneous pathology

UV exposure increases many transcription factors, which may be acting synergistically to improve the immune system. Vitamin D is a result of UV exposure, but it may not be the only molecule utilized for health benefits. Mendelian randomization finding associated genotypes with low 25(OH)D with increased mortality for all diseases except cardiovascular events. This shows that mediators, other than vitamin D, help with cardiovascular disease [20].

Low levels of vitamin D result in higher mortality and disease, and there is an inverse relationship between levels of vitamin D and cardiovascular events and cancer. Supplementation was not indicated to protect against these diseases indicating endogenous vitamin D from UV exposure is the most efficacious protection method. Further studies by Garland et al. indicate that there are twice as many age-adjusted death rates when vitamin D levels are below 22 nmol/L as compared to levels greater than 125 nmol/L. Lindqvist et al. demonstrated that sun avoidance and smoking were of the equal magnitude of risk. Growing evidence shows lower rates of cardiovascular disease and blood pressure incidence with higher sunlight exposure [20].

Hypertension is generally considered to occur due to high levels of vasoconstrictor molecules such as angiotensin and epinephrine, but it is also due to low levels or impaired function of vasodilators like NO. A large amount of NO is generated and contained within the skin. It is hypothesized that upon UV exposure, these molecules are released and help relax the vasculature; thus, lowering or maintaining blood pressure. Studies demonstrate that in healthy humans, 20 J/cm^2^ of sunlight and around 30 minutes of mid-day light in locations similar in latitude to Florida increase NO release and relax vessels [20]. However, high levels of NO can also lead to carcinogenesis. DNA damage through ROS and other molecules leading to damage, such as NO, are drawbacks to UV exposure. At high levels, NO loses its efficacy for vasodilation and can combine with ROS to form peroxynitrite. Peroxynitrite induces peroxidative and nitrosative damage to DNA, nitrosylation of tyrosine residues, and can initiate lipid peroxidation. Furthermore, peroxynitrite activates poly(adenosine diphosphate-ribose) polymerase, which converts NAD^+^ to nicotinamide and ADP-ribose, which depletes adenosine triphosphate (ATP) and NAD^+^ in the cells [25].

To quantify vitamin D production in patients with end-stage renal disease, patients using dialysis were exposed to UV lamps, and serum 25(OH)D levels were measured. Within seven weeks, whole-body radiation exposure increased 25(OH)D levels from 6 ng/mL to 36 ng/mL, then to 86 ng/mL over the next six months. Partial UV exposure just on the patients’ legs increased 25(OH)D from 25 ng/mL to 39 ng/mL. In both experiments, 25(OH)D remained stable after the course of UV treatment. As documented in healthy individuals, only 15% of body surface area is needed to generate high-normal serum 25(OH)D levels, which is comparable to whole-body exposure. During the study, other measurements were obtained, which highlighted the benefits of higher UV-induced vitamin D levels. A significant decrease in sitting blood pressure levels was indicated. A total of 50% of chronic kidney disease patients also have hypertension or other cardiovascular morbidities. By utilizing UV light to induce higher vitamin D levels, they may be able to reduce cardiovascular complications. Patients in the study were also able to increase their workload by bicycle ergometer test by 6%, increase oxygen uptake by 11%, and reduce lactic acid by 9%. Electrocardiography measurements taken indicated a 20% increase in RR interval time and a standard deviation for the beat-to-beat difference of 25%. This suggests that UV exposure improved cardiovascular health as equally as performing cardiovascular endurance training. Vitamin D metabolites have been used in chronic kidney disease patients to decrease parathyroid hormone levels [4].

Vitamin D regulates and suppresses the inflammatory response of respiratory epithelial cells and macrophages. Recent evidence has suggested vitamin D supplementation can reduce death through cathelicidin and defensin induction. Since vitamin D production is mostly produced from the skin, phototherapy disruptions during the pandemic may increase COVID-19 morbidity. Furthermore, the expression of DPP-4 and CD26 receptors, which interact with the spike protein of COVID-19 viruses, is reduced in vivo once a vitamin D deficiency resolves. In these contexts, vitamin D plays an important role in the development and reduction of severe COVID-19 infections [23].

A study was conducted that utilized 88 countries that could provide accurate data about COVID-19 incidence and mortality. Data from May 11, 2020, compared Ireland to Singapore due to their similar population sizes of 4.9 million versus 5.3 million, respectively. The total number of positive cases was also similar (23,125 and 23,822). The cases per million people were close as well (4,685 per million versus 4,072 per million). Results showed Ireland’s COVID-19 death rate was 297 per million, but Singapore’s was only 4 per million. This study found a strong correlation between latitude and the COVID-19 death rate, although serum vitamin D was not measured. It can be inferred that lower-latitude locations offer higher UV radiation for the cutaneous production of vitamin D. This study is observational and should be viewed with scrutiny. Many factors result in the mortality rates of COVID-19, not just vitamin D status or proximity to the equator. This study allows further questioning into more studies to evaluate vitamin D’s role with COVID-19 mortality, but no conclusions can be made without serum 25(OH)D levels [26].

A recent study showed that temperature and mean UVR (J/m^2^) show no influence on the transmissivity of COVID-19. However, additional studies have indicated that increased levels of plasma pro-inflammatory cytokines such as IL-6 correlate to disease severity. UVR may play a critical role in this pathway by inducing immune system suppression. In critically ill patients with COVID-19, tocilizumab has been used to reduce cytokine storm progression via blocking IL-6. Vitamin D potentially plays a role in COVID-19 mortality. Countries below 35 degrees of latitude had lower mortality to COVID-19 compared to northern countries. Several studies have shown how vitamin D regulates and suppresses the inflammatory response of respiratory epithelial cells and macrophages. Recent evidence has suggested vitamin D supplementation can reduce death through cathelicidin and defensin induction. Since vitamin D production is mostly produced from the skin, phototherapy disruptions during the pandemic may increase COVID-19 morbidity [23].

Vitamin D has been reported to induce apoptosis in many cancer cell lines, such as the prostate, breast, and colon. Recently, studies have indicated vitamin D has protective benefits on the skin post-UV exposure. One study conducted by Dixon et al. demonstrated protection against UV-induced cell death with 1,25D concentrations as low as 0.01 nM-10 nM in fibroblasts and 1 nM-100 nM in keratinocytes. Other groups have noted that a higher concentration, up to 1 μM of vitamin D, is necessary to protect against UV-induced apoptosis. This group did not notice cytotoxicity with this concentration, but other groups have reported cytotoxicity. More studies are needed to confirm safe levels of vitamin D, which does not generate cytotoxicity but protects against UV radiation [25].

Within the United States and Europe, vitamin D status among adolescents has been widely researched. However, more recent data indicates hypovitaminosis D is re-emerging. Rickets' incidence has been increasing over the past two decades and mostly affects non-Caucasian children. Dietary vitamin D is found in oily fish, cod liver oil, egg yolks, organ meats, and liver. Vegetables, cereals, and fruits contain minimal amounts of vitamin D. Human milk contains vitamin D but is influenced by maternal skin color, latitude, UV exposure, clothing, and season. However, supplementing 4,000 IU of vitamin D3 daily improved the mothers’ serum 25(OH)D to over 30 ng/mL and transferred enough vitamin D3 to meet their infant’s requirements [27].

With the reappearance of rickets within North America, Europe, and the UK, new studies were conducted to measure incidence. A UK study reported an incidence of 7.5 per 100,000 in newborn to five-year-old children with the highest incidence of African children at 95 per 100,000. The “Global Consensus Recommendations on Prevention and Management of Nutritional Rickets” has classified vitamin D status based on serum 25(OH)D levels. These categories include sufficient (>20 ng/mL), insufficient (12-20 ng/mL), deficient (<12 ng/mL), and toxicity (>100 ng/mL with hypercalcemia, hypercalciuria, and low parathyroid hormone level) [27].

Age is a risk factor for vitamin D deficiency. Newborn vitamin D status is solely dependent on maternal vitamin D status or commercially produced baby formula content. Low maternal vitamin D levels during pregnancy have adverse neonatal outcomes, such as preterm birth and small for gestational age. Newborns primarily need vitamin D from exogenous sources. As the child grows, hypovitaminosis D is attributed to a poor diet. In obese children, adipose stores of vitamin D do not properly release upon hypovitaminosis D status, but no evidence supports that bone health is affected. A sedentary lifestyle is a risk factor for vitamin D deficiency. With the emergency of high-speed internet, mobile smartphones, video games, and television, a focus from children playing outside to staying indoors increases the risk of vitamin D deficiency and obesity [27].

Seasonal infections like influenza usually present around wintertime. This is also when serum vitamin D levels will be at their lowest for most people who do not live within 30 degrees of the equator. Several observational and interventional studies have demonstrated this effect. For healthy adults living in New England, they halved their risk of acute viral respiratory tract infections if their serum 25(OH)D was around 38 ng/mL. In Japan, children who ingested 1200 IU of vitamin D3 daily for four months during the winter reduced their risk of developing influenza by 42%. Furthermore, infants whose 25(OH)D3 levels were >30 ng/mL were six times less likely to contract respiratory syncytial virus than infants whose 25(OH)D3 levels were <20 ng/mL [5].

Public health guidelines and recommendations

The Food and Drug Administration (FDA) set a recommended daily allowance of vitamin D at 400 IU [28]. In 1999, Vieth suggested that adults needed to consume at least 1,000 IU to protect against bone fractures. The tolerable upper limit is set at 2,000 IU; however, studies showing vitamin D overdoses or toxicity are poorly constructed and funded. On a sunny day, the skin can generate 10,000 IU of vitamin D just from UV light exposure [1]. 

Breastfeeding mothers can pass vitamin D through their breastmilk, but not in large quantities. It has been suggested that infants with minimal sun exposure need 400-500 IU of vitamin D daily. Maternal vitamin D supplementation from ingested vitamin D or through cutaneous manufacturing of vitamin D is needed in upwards of 6,000 IU per day to maintain their offspring’s 25(OH)D levels at >50 nmol/L [8].

A study following young Americans suggests they may need more vitamin D than previously recommended. By taking 2,000 IU/day early in life, the risk of type I diabetes mellitus may be significantly reduced. Health agencies have recommended against sun exposure to minimize skin cancer [10]. Public health officials advocate for a sun protection factor and clothing usage, which lowers UV exposure. Few campaigns take the benefits of endogenous vitamin D into account when advocating for “sun avoidance". With many sunscreens protecting against UVA and UVB radiation, more studies indicating the benefits from safer UV exposure are needed [1]. Consequently, serum vitamin D levels across the United States are low. In U.S. children, it is estimated that only half are generating >1,000 IU of vitamin D daily [10].

In the UK, the SunSmart program was introduced in 2002. Its goal was to increase knowledge of skin cancers, increase awareness of actions to prevent skin cancers, and influence mindsets about the sun and how to protect against it. With melanoma rates rising quickly, public health officials advocated for more sun protection when necessary. Data gathered during the SunSmart campaign indicated residents of the UK greatly enjoy basking in the sun. Some quotes from the program from residents include, “skin cancer’s the last thing I am thinking about” and “you do feel good spending a day lying out in the sun.” Many believed a sunburn was required to induce tanning and that covering the body in protective measures would inhibit tanning [29].

Quantitative research through the National Statistics Omnibus Survey demonstrated that adolescents were the most difficult group of people to influence. Their desire to tan was also the highest with 70% of adolescents aged 16-24 years planned on tanning while on vacation. In the study, 77% indicated sunscreen was the primary method of skin protection, but only one-third stated they used it. More public health initiatives are needed due to the sporadic nature of UV exposure in the UK. Many individuals try to “make the most” of their vacation and absorb as much UV exposure as possible; however, with melanoma incidence primarily from repeated sunburn, better initiatives are needed to educate the public about sun exposure and protection. There are also unanswered questions regarding the low 25(OH)D levels within the UK’s population despite a rising incidence of melanoma [29].

In a cross-sectional study conducted in Australia, participants given a telephone survey answered several questions about vitamin D and the sun. A majority, 84%, of participants heard of vitamin D, mostly from the media, physicians, or pharmacists. They also indicated that vitamin D is beneficial for skin health, bone health, and eyesight. Finally, 82% of men and 90% of women surveyed agreed that vitamin D can be obtained through sun exposure. Nearly one-third of participants thought light-skinned individuals needed 30 minutes of exposure per day during the summer. When asked if they thought they were in danger of not getting enough vitamin D when protecting their skin from UV exposure, 32% agreed and 16% were unsure. Around 21% of individuals indicated they had changed their sun exposure habits and limited their intake of UV radiation. Out of those, 20% said they did so upon hearing advice from a health professional. Over three-quarters thought their children were getting adequate sun exposure, and 31% believed their children needed only 30 minutes of exposure per day during the summer. Since this study was conducted via telephone and was a survey, it is potentially flawed and should be read with scrutiny. However, this survey indicates a vast majority of the population knows some information about vitamin D and its health benefits [13].

Exogenous and endogenous vitamin D are both beneficial to health. Limited food products are vitamin D fortified, which makes adequate amounts of vitamin D difficult to obtain through diet alone. In one study, only 5% of the body’s skin surface was needed to produce adequate amounts of vitamin D. The backs of the hands and face account for greater than 5% of the body's surface area. Many sun protection campaigns facilitate the idea of limiting sun exposure to less than 15 minutes per day; however, a study in Boston indicates a light-skinned individual will achieve maximum vitamin D production within five minutes. The rest of the time spent outdoors leads to UV-induced skin damage [6].

Supplementation of vitamin D, in recent studies, is shown to be safe up to 4,000 IU daily. With an upper limit of 10,000 IU daily for both cutaneous and dietary vitamin D, the likelihood of hypervitaminosis D and hypercalciuria is low. Generating high amounts of vitamin D from UV exposure is unlikely due to the high amount of sunlight required. The amount of UV exposure needed to induce such high serum vitamin D levels is equivalent to one minimal erythema dose over greater than 27% of the total body surface area [6].

Studies examining proper 25(OH)D levels indicate there may not be a relative “normal.” Before modern civilization, humans spent much of their time outdoors soaking in UV radiation with no commercial sunscreen. It has been suggested that a baseline for proper serum vitamin D amounts should be compared to lifeguards. Their average serum 25(OH)D was 161 nmol/L, whereas the general population was 68.3 nmol/L. However, lifeguards in the study with 25(OH)D levels at 148 nmol/L exhibited hypercalciuria indicating hypervitaminosis D. A safer level to strive for was suggested to be 140 nmol/L [6]. Advising patients to increase vitamin D levels is difficult to quantify. Body surface area, sunny days per year, latitude, sun protection factor, and exposure time are all factors that aid and inhibit vitamin D status. Studies have shown that after CMM diagnosis vitamin D levels decrease, but recurrent skin cancer risk increases. Encouraging skin cancer patients to safely increase vitamin D levels through UV radiation may reduce further skin cancer diagnoses and help improve their overall health. More research is needed to identify the amount of sunlight needed to safely increase 25(OH)D [22].

## Conclusions

More research is needed to determine how much vitamin D is needed to sustain healthy living. With dozens of factors influencing how much vitamin D is generated from UV exposure, there is not a one-size-fits-all approach to recommendations. Patients with vitamin D levels below 16 ng/mL were at greater risk of morbidity and mortality. Increasing vitamin D levels above 20 ng/mL have been shown to improve disease outcomes. Since many cancer and high-risk disease prognoses benefit from higher serum vitamin D levels, accurate vitamin D monitoring may help reduce disease burden and prolong patient lives. Toxicity is rare due to the self-regulating mechanisms the kidney uses when generating UV-induced vitamin D. This is mainly determined when there is evidence of hypercalciuria and hypercalcemia with hyperparathyroid hormone-like symptoms. When no other disease pathology is present, reducing UV exposure or supplementation will lower calcium levels. Vitamin D’s inherent nature of being self-regulating and safe should encourage the public to increase UV exposure safely and seek regular skin exams. By avoiding sunburn and wearing high-quality sunscreen, higher levels of vitamin D can be achieved while minimizing the risks associated with UV exposure. When UV light is not abundant, vitamin D supplementation may also benefit health.
